# Biogenic Nanoparticle‒Chitosan Conjugates with Antimicrobial, Antibiofilm, and Anticancer Potentialities: Development and Characterization

**DOI:** 10.3390/ijerph16040598

**Published:** 2019-02-19

**Authors:** Muhammad Bilal, Yuping Zhao, Tahir Rasheed, Ishtiaq Ahmed, Sherif T.S. Hassan, Muhammad Zohaib Nawaz, Hafiz M.N. Iqbal

**Affiliations:** 1 School of Life Science and Food Engineering, Huaiyin Institute of Technology, Huaian 223003, China; zhaoyuping@hyit.edu.cn; 2 School of Chemistry and Chemical Engineering, State Key Laboratory of Metal Matrix Composites, Shanghai Jiao Tong University, Shanghai 200240, China; masil@sjtu.edu.cn; 3 School of Medical Science, Menzies Health Institute Queensland, Griffith University (Gold Coast campus), Parklands Drive, Southport, QLD 4222, Australia; i.ahmed@griffith.edu.au; 4 Department of Natural Drugs, Faculty of Pharmacy, University of Veterinary and Pharmaceutical Sciences Brno, Palackého tř. 1946/1, 612 42 Brno, Czech Republic; sherif.hassan@seznam.cz; 5 Department of Computer Science, Center for Advanced Studies in Agriculture and Food Security, University of Agriculture, Faisalabad 38040, Pakistan; zohaib_binm@yahoo.com; 6 Tecnologico de Monterrey, School of Engineering and Sciences, Campus Monterrey, Ave. Eugenio Garza Sada 2501, Monterrey, N.L. CP 64849, Mexico

**Keywords:** *Convolvulus arvensis*, AgNPs, chitosan, antibacterial, antibiofilm, anticancer

## Abstract

In the 21st century, with ever-increasing consciousness and social awareness, researchers must tackle the microbial infections that pose a major threat to human safety. For many reasons, the emergence/re-emergence of threatening pathogens has increased and poses a serious challenge to health care services. Considering the changing dynamics of 21st-century materials with medical potentialities, the integration of bioactive agents into materials to engineer antibacterial matrices has received limited attention so far. Thus, antimicrobial active conjugates are considered potential candidates to eradicate infections and reduce microbial contaminations in healthcare facilities. In this context, eco-friendly and novel conjugates with antimicrobial, antibiofilm, and anticancer potentialities were developed using biogenic silver nanoparticles (AgNPs) from *Convolvulus arvensis* (*C. arvensis*) extract and chitosan (CHI). A range of instrumental and imaging tools, i.e., UV-Vis and FTIR spectroscopy, scanning electron microscopy (SEM), transmission electron microscopy (TEM), energy-dispersive spectroscopy (EDX), and X-ray diffraction (XRD), were employed to characterize the freshly extracted *C. arvensis* AgNPs. Biogenic AgNPs obtained after a 24-h reaction period were used to engineer CHI-based conjugates and designated as CHI‒AgNPs1 to CHI‒AgNPs5, subject to the *C. arvensis* AgNPs concentration. After the stipulated loading period, 92% loading efficiency (LE) was recorded for a CHI‒AgNPs3 conjugate. Gram+ and Gram- bacterial isolates, i.e., *Staphylococcus aureus*, and *Escherichia coli*, were used to test the antibacterial activities of newly developed CHI‒AgNPs conjugates. In comparison to the control sample with bacterial cell count 1.5 × 10^8^ CFU/mL, a notable reduction in the log values was recorded for the CHI‒AgNPs3 conjugate. The antibiofilm potential of CHI‒AgNPs conjugates was tested against *Pseudomonas aeruginosa*. Moreover, the CHI‒AgNPs3 conjugate also showed substantial cytotoxicity against the MCF-7 (breast cancer) cell line. In summary, the newly engineered CHI‒AgNPs conjugates with antibacterial, antibiofilm, and anticancer potentialities are potential candidate materials for biomedical applications.

## 1. Introduction

Materials at the nanoscale are evolving and are of immense interest for researchers due to a range of applications including medication, pigments, catalysts, electronic displays, electric batteries, sensors, foodstuff, farming, and construction [1,2,3,4,5,6]. The current advancements at the nanoscale have made it possible to engineer nanoconstructs of tuned sizes [7,8]. AgNPs are widely used in medical fields such as surgical schemes, wound bandaging, and stents [9,10,11,12,13,14]. The characteristics of innovative nanomaterials are largely size- and shape-dependent. So enormous efforts have been made to propose methods for the synthesis of controlled surface properties of size and shape for as-synthesized metal nanoparticles [15]. The antibacterial activity of silver-based nanoparticles (AgNPs) depends on their size and stability in solution [16]. Colloidal solutions of AgNPs often aggregate and suppress antibacterial activity [17,18]. Consequently, stable AgNPs are critical for useful biocidal-material applications [19].

Various physical and chemical techniques can prepare AgNPs, including: sonochemical [20], continuous-flow process [21,22], phyto-synthesis [23], laser-mediated technique [24], electrochemical [25], solution reduction [26,27,28], thermal decomposition [29], physiochemical methods [30], microwave technique [31], photochemical production [32], solvothermal [33], and wet chemical [34]. Wet chemical methods generally achieve mass production of monodispersive metal nanoparticles in a cheap and convenient way. Syntheses of AgNPs of varied shapes and sizes have been performed through liquid-phase reduction of silver nitrate [35,36]. The excessive use of harsh chemicals in metal-based nanoparticles preparation suppresses the utility of nanoparticles in biomedical fields [37,38]. The abovementioned issues related to the synthesis of metal-based particles at the nanoscale in the presence of toxic and harsh chemicals have urged researchers to design new, useful, and eco-friendly methods for the production of highly stable metal nanoparticles [39,40]. Therefore, alternative methods to govern the size and shape of the metal nanoparticles have tended to be prepared via green synthesis, which uses natural plants or bio-organisms [41,42,43,44].

Green synthesis eliminates the use of toxic chemicals in the preparation of silver nanoparticles. Aggregation of silver onto AgNPs is avoided by loading them onto a support surface, resulting in AgNPs-loaded materials or conjugates. These materials can be reprocessed for reuse as other colloidal solutions have the limitation of recycling. Binding of the nanoparticle to support materials is crucial as binding materials play a significant role in the strong adherence of depositing nanoparticles [45]. Chitosan (a deacetylated derivative of chitin) contains amino groups with high nitrogen content and has vast applications in medicine, environment, the food sector, agricultural settings, and the makeup industry [46,47], due to its high biocompatibility, biodegradability, and low/no toxicity along with antimicrobial properties [48,49].

In this study, *Convolvulus arvensis* was used for the biogenic synthesis of AgNPs. Different instrumental and imaging techniques were used to characterize the newly developed biogenic *Convolvulus arvensis* AgNPs. The optimally yielded AgNPs were used to develop GA-assisted CHI‒AgNPs conjugates with biomedical potentialities. The research was conducted to determine experimental aspects of the combined effects of AgNPs and CHI in CHI‒AgNPs conjugates for their antibacterial, antibiofilm, and anticancer potentialities.

## 2. Material and Methods

### 2.1. Chemicals and Reagents

All the chemicals and reagents used in this study were of analytical laboratory standard with purity >98%. Antibiotic (penicillin‒streptomycin) solution, chitosan (MW 100-300 kDa with 82% degree of deacetylation), glutaraldehyde (GA), silver nitrate (AgNO_3_), potassium bromide (KBr), Dulbecco’s modified Eagle’s Medium (DMEM), fetal bovine serum (FBS), 3-(4, 5 -dimethylthiazol-2-yl)-2, 5-diphenyl tetrazolium bromide (MTT) were obtained from the local distributors of Sigma-Aldrich. All other chemicals/reagents were used without further purification unless otherwise specified.

### 2.2. Convolvulus Arvensis Extract Preparation

Fresh leaves of *Convolvulus arvensis* (*C. arvensis*) were collected and washed (3–4 times) with tap water to remove dust. Following the stipulated washing, the *C. arvensis* leaves were dried overnight at 28 ± 2 °C on Whatman No. 1 filter paper (Sigma-Aldrich). An electrical blender was used to grind the overnight dried leaves to a fine powder. The resultant powder was stored in airtight polyethylene bags to keep it moisture-free. To prepare an extract solution, the freshly obtained *C. arvensis* leaf powder (one gram) was macerated three times with 100 mL of absolute methanol at 28 ± 2 °C. The rotary evaporator at 45 ± 2 °C was used to eliminate the excessive solvent, followed by lyophilization and stored at 4 °C for subsequent experimental analysis.

### 2.3. Microbial Cultures and Cell Line

Three bacterial strains, i.e., *Staphylococcus aureus* (*S. aureus*), *Escherichia coli* (*E. coli*), and *Pseudomonas aeruginosa* (*P. aeruginosa*), were used in this study. *S. aureus* and *E. coli* were used to test antibacterial acidity. *P. aeruginosa* was used to validate the viability (reaffirm the antibacterial potential) and antibiofilm activity. To develop a stock inoculum, the collected cultures were grown overnight in a sterile nutrient broth (50 mL) at 30 °C and 120 rpm. After a stipulated period of incubation, i.e., 18 h, each culture was diluted appropriately to yield an initial bacterial count, i.e., 1.5 × 10^8^ CFU/mL (control value). The MCF-7 cell line (breast cancer) was used for cytotoxicity analysis by MTT assay.

### 2.4. Biogenic AgNPs Preparation

The freshly prepared methanolic leaf extract of *C. arvensis* was used to synthesize AgNPs using various concentrations of AgNO_3_, i.e., 20, 50, and 100 mM. The reduction of AgNO_3_ solution into Ag^+^ ions was initiated by mixing a known quantity of AgNO_3_, and freshly prepared extract of *C. arvensis* leaves. The above mixture was stirred continuously for 10 min, followed by 2 h incubation at 28±2 °C. The reaction termination was observed with a color change from light yellow to blackish-brown. UV–Visible spectroscopy was used to confirm the bio-reduced sample by taking an absorbance scan at the wavelength between 200 and 800 nm. To further purify the *C. arvensis* AgNPs, the reduced mixture was centrifuged at 4000× *g* for 15 min and washed with deionized water. The obtained sample was oven-dried for 48 h at 50 °C to constant weight and stored at 4 °C for characterization and CHI‒AgNPs conjugate preparation.

### 2.5. UV-Vis Spectral Analysis

A Shimadzu UV-visible spectrophotometer (UV-7504, Xinmao, Shanghai, China) was used to record the absorbance of *C. arvensis* AgNPs containing extract. A quartz cell (1.0 cm path length) was used to record the absorbance of 300 µL of AgNPs at the wavelengths from 200 to 800 nm. The λ_max_ values were noted from 0 h to each hour until 24 h by taking an aliquot from the same mother liquor.

### 2.6. Instrumental and Imaging-Based Evaluation of *C. arvensis* AgNPs

The extracted AgNPs were characterized using various analytical and imaging techniques. FT-IR spectra of newly developed *C. arvensis* AgNPs were recorded at a wavelength range of 4000‒500 cm^−1^ with 64 scans at a resolution of 4.0 cm^−1^ (PerkinElmer Spectrum 100 FTIR spectrometer, PerkinElmer Inc., Waltham, MA, USA). The samples were pressed with dried KBr and analyzed as a pellet and assigned peak numbers. An X-ray diffractometer (D8 Advance X-ray diffractometer, Bruker AXS, Karlsruhe, Germany) was used to record the X-ray diffractogram of *C. arvensis* AgNPs using the following working conditions, i.e., 40 kV voltage, 30 mA current, and 2θ° angles. SEM (JSM 7800F, JEOL Ltd., Tokyo, Japan) with an accelerating voltage of 5 kV was used to analyze the surface morphology of *C. arvensis* AgNPs. The test samples (*C. arvensis* AgNPs) were mounted onto the surface of silicon chips and coated with gold using a gold sputtering device. The operating conditions were as follows: accelerated voltage (5 kV), pressure (7 × 10^−2^ bar), and deposition current (20 mA). An energy-dispersive X-ray (EDX) detector was used to record the elemental profile of newly developed *C. arvensis* AgNPs. For TEM analysis, *C. arvensis* AgNPs were placed onto the carbon-coated copper grid and envisaged using TEM (Tecnai G2 Spirit Biotwin FEI Company, Hillsboro, OR, USA).

### 2.7. Preparation of CHI‒AgNPs Conjugates

The one-pot synthesis approach was used to develop CHI‒AgNPs conjugates with different AgNPs concentrations. Briefly, a CHI solution (0.5%, w/v) was dispersed ultrasonically and sequentially added dropwise in 5.0% (w/v) acetic acid solution over 1 h with stirring at 28 ± 2 °C. Following that, different concentrations of *C. arvensis* AgNPs, i.e., 1–5% (w/v), each separately, were extruded drop by drop into 20 mL chitosan solution under continuous stirring at 120 rpm. The above mixture was activated using 0.5% (w/v) GA solution (freshly prepared within a 50 mM Na‒malonate buffer of pH 4.5) for another 2.0 h and poured into a sterile, labeled Petri plate followed by 24 h incubation in a hot air oven at 50 °C to develop CHI‒AgNPs conjugates. The resultant GA cross-linked CHI‒AgNPs conjugates were recovered and washed three times with distilled water. Subject to the *C. arvensis* AgNPs concentration, the obtained conjugates were designated CHI‒AgNPs1, CHI‒AgNPs2, CHI‒AgNPs3, CHI‒AgNPs4, or CHI‒AgNPs5.

Equation (1) was used to calculate the percent loading efficiency (%LE):

(1) $$Loading\;efficiency\;\left(\%\right)=\frac{Wf-Wi}{Wi}\times 100,$$

where *Wf* = final dry weight and *Wi* = initial weight (chitosan without AgNPs).

### 2.8. Evaluation of Antibacterial Activity

The antibacterial activities of the newly developed pristine AgNPs and AgNPs-loaded CHI‒AgNPs conjugates were evaluated against *S. aureus* and *E. coli*. All CHI‒AgNPs conjugates were sanitized for half an hour (30 min) at 90 °C. A freshly prepared suspension containing approximately 1.5 × 10^8^ CFU/mL of *S. aureus* and *E. coli* was spread onto the surfaces of the test conjugates and incubated at 30 °C for 24 h [50]. The control sample was incubated in the presence of sterile nutrient broth (15 mL), while the test conjugates were incubated in the presence of 15 mL phosphate buffer. At the end of 24 h incubation, both control and test samples were washed using 50 mL phosphate buffer (pH 7.0). The washed suspension was used to record the cell viability as CFU/mL using a plate counter agar. Equation (2) was used to calculate the log reduction to assess the antibacterial activity [50,51,52]. A 2-log reduction was considered a threshold to report antibacterial activity [52].

(2) Log reduction=Log CFU Control sample−Log CFU Treated sample

### 2.9. Antibiofilm Viability Impact Assay

The antibiofilm viability of the CHI‒AgNPs conjugates was tested against *P. aeruginosa*. Briefly, a freshly prepared bacterial suspension (1.5 × 10^8^ CFU/mL) was spread onto the surfaces of the test conjugates and incubated for 72 h, under still culture environment to allow the viable bacteria to adhere onto the test surface. Samples without AgNPs or CHI‒AgNPs were trialed as a control biofilm. After the stipulated incubation period (72 h), a sterilized phosphate buffer saline was used to wash the test conjugates to remove the free floating and weakly bounded cells. It was observed that test surfaces with strong antibacterial potential did not allow the bacteria to form a mature/compact biofilm. Following washing, the live and dead cells distribution was accessed by staining with 100 µL LIVE/DEAD *Bac*Light Bacterial Viability Kit (ThermoFisher Scientific, Waltham, MA, USA). The images were recorded via confocal laser scanning microscopy (CLSM).

### 2.10. Evaluation of Cytotoxicity by MTT Assay

Both control (AgNPs alone) and CHI‒AgNPs conjugates were subjected to cytotoxicity evaluation by MTT assay. For this purpose, a MCF-7 (breast cancer) cell line was used to access the anticancer potential, as reported by Rasheed et al. [27]. Briefly, MCF-7 cells were grown for 24 h at 37 °C in 96-well microtiter plates (pre-inoculated with AgNPs alone and CHI‒AgNPs conjugates) using a DMEM that was additionally supplemented with 10% of FBS. After 24 h incubation, the DMEM was removed. The MCF-7 cells were again incubated for 4 h at 37 °C in the presence of 20 μL of MTT (5 mg/mL in PBS) supplemented fresh medium. Following that, DMSO (150 μL/well) was used to solubilize the formazan crystals resulting from the mitochondrial reduction of MTT. Finally, the absorbance was recorded at 570 nm (2300 EnSpire Multilabel Plate Reader, Perkin Elmer). Equation (3) was used to calculate the percent MCF-7 viability:

(3) $$\mathrm{Cell}\;\mathrm{viability}\;\left(\%\right)=\frac{\mathrm{Absorbance}\;\mathrm{of}\;\mathrm{test}\;}{\mathrm{Absorbance}\;\mathrm{of}\;\mathrm{control}}\;\times 100.$$

## 3. Results and Discussion

### 3.1. UV-Vis Spectral Analysis

**Figure 1:**
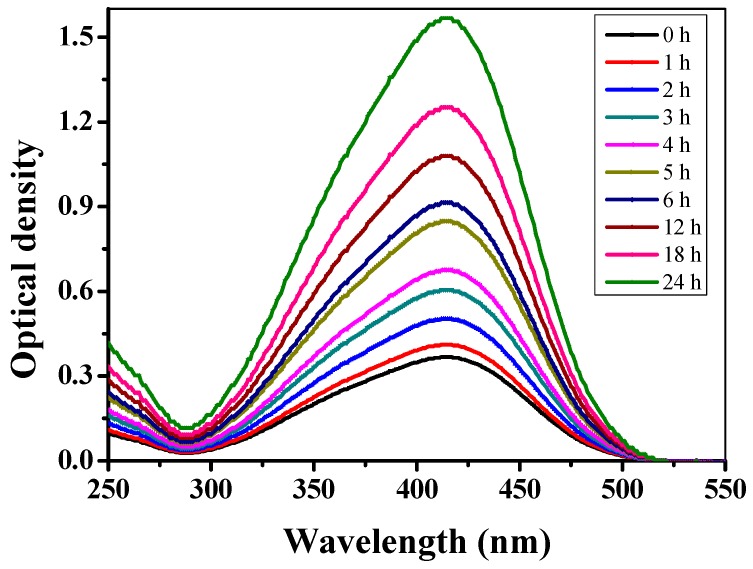
UV-Vis spectral analysis of control and freshly extracted *C. arvensis* AgNPs.

Figure 1 illustrates a UV-Vis spectroscopic profile of in-house extracted *C. arvensis* AgNPs at various time intervals (0‒24 h). A visible color change from colorless to blackish-brown was observed upon the addition of freshly prepared *C. arvensis* leaves extract to AgNO_3_. The intensity of the color is proportional to the reaction time, which is due to the reduction of a silver ion from Ag^+^ to Ag^0^ accompanied by the Surface Plasmon Resonance (SPR) phenomenon [53]. On the contrary, no color change was observed in the samples without the addition of *C. arvensis* extract. As shown in Figure 1, a broader peak around 420 nm demonstrated the surface plasma resonance of AgNPs. Time-course analysis of *C. arvensis* extract and AgNO_3_ revealed a clear interaction between then, which led to the accumulation of *C. arvensis* AgNPs. The characteristic peak at around 420 nm of λ_max_ was quite small in the samples extracted within the first couple of hours. However, samples extracted at and after 12 h showed a notable intensification in the peak at 420 nm [54], which is possibly due to the SPR phenomenon.

### 3.2. Instrumental and Imaging-Based Evaluation of *C. arvensis* AgNPs

**Figure 2:**
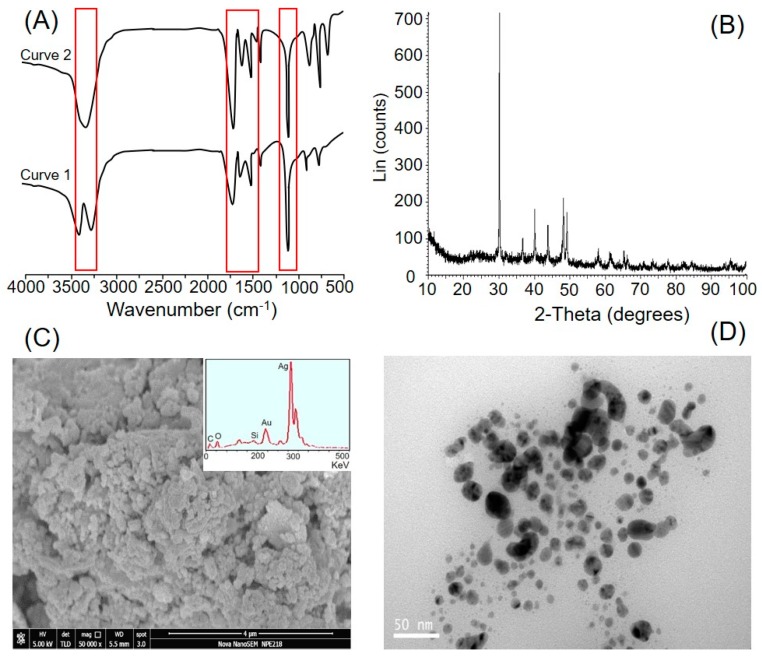
Characterization of *Convolvulus arvensis* AgNPs: (**A**) Fourier transform infrared spectroscopy (FT-IR) spectrum, (**B**) X-ray diffraction (XRD) spectrum, (**C**) scanning electron microscopy (SEM), and (**D**) transmission electron microscopy (TEM).

The FTIR spectrum was recorded to confirm the formation of *C. arvensis* AgNPs further, owing to the interactions between *C. arvensis* extract and AgNO_3_. Furthermore, the FTIR profile revealed the potential functional moieties responsible for the reduction of a silver ion from Ag^+^ to Ag^0^ accompanied by the excitation of Surface Plasmon Resonance (SPR) phenomenon (evidenced by UV-Vis spectral analysis) and capping of bio-reduced AgNPs prepared from *C. arvensis* extract. The FTIR spectral profile of the *C. arvensis* extract showed characteristic peaks at 1115, 1588, 1640, 1748, and 3320 cm^−1^ (Figure 2A, curve 1). The bands at 1115 and 1388 cm^−1^ correspond to C‒O-‒H stretching and C‒H bending, respectively. A band at 1588 cm^−1^ can be assigned to the vibrational stretching of the C‒C skeleton [55]. The characteristic band at 1748 cm^−1^ corresponds to the vibrational stretching of carbonyl functional groups available in aldehydes, ketones, and carboxylic acid moieties. The presence of an intense and broader band at 3320 cm^−1^ refers to the characteristics of hydroxyl functional groups available in alcohols and phenolic constituents of the *C. arvensis* extract. The FTIR spectral profile of AgNPs exhibits characteristic peaks at 1640, 1748, and 3320 cm^−1^ (Figure 2A, curve 2). According to the literature, the water-soluble fractions of plant extracts contain large amounts of terpenoids with a larger contribution of citronellol and geraniol along with a smaller fraction of linalool [56]. The appearance of a peak at 1748 cm^−1^ suggests the possible involvement of terpenoids in the reduction of a silver ion from Ag^+^ to Ag^0^; the terpenoids are oxidized to carbonyl groups that result in a characteristic band at 1748 cm^−1^. The broadness in the band at 1640 cm^−1^ corresponds to the capping of AgNPs [57].

Figure 2B presents the XRD profile of *C. arvensis* AgNPs obtained after 24 h of the reaction period. The appearance of sharp 2θ peak values confirmed the AgNPs’ nanosize [58]. As reported in an earlier study, some Bragg reflections may be indexed by the fcc structure of silver, which suggests strong X-ray scattering centers in the crystalline phase [57]. Similar observations have been reported in other XRD studies with 15 nm crystallite size of AgNPs [28,54,59,60,61]. The SEM and TEM photographs of the newly extracted *C. arvensis* AgNPs are shown in Figure 2C,D, respectively. It is clear from the SEM and TEM images that *C. arvensis* AgNPs agglomerate and fuse loosely. EDX analysis was performed to investigate the elemental composition of *C. arvensis* AgNPs, which indicates the presence of metallic silver in high quantities (Figure 2C). Moreover, traces of other elements, i.e., oxygen (O), silicon (Si), and carbon (C), as impurities, were also observed. As evident from the TEM analysis, most of the *C. arvensis* AgNPs were spherical with an average diameter in the range of 45 nm.

### 3.3. CHI‒AgNPs Conjugates

**Figure 3:**
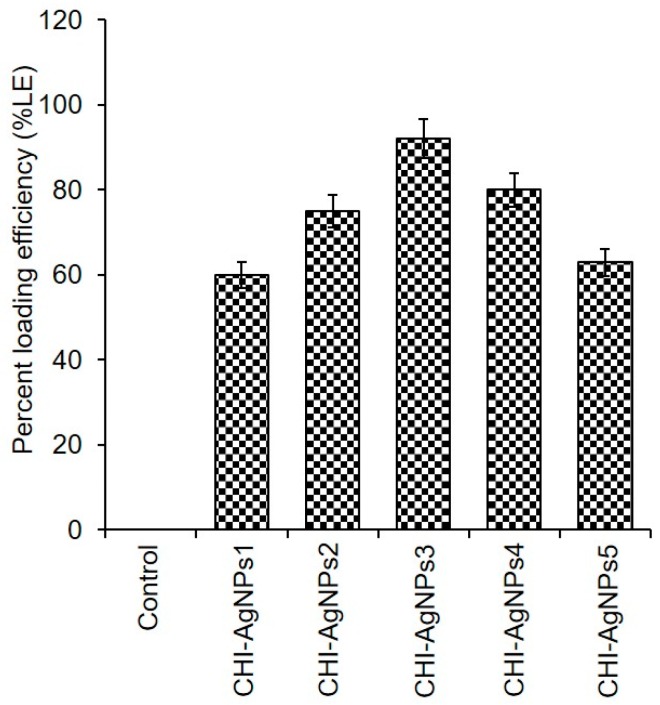
The percent loading efficiency (LE) of AgNPs into the CHI-based conjugate.

The GA-assisted CHI‒AgNPs conjugates were prepared using different concentrations of newly extracted *C. arvensis* AgNPs. The %LE of AgNPs into/onto the CHI-based conjugate, i.e., CHI‒AgNPs1, CHI‒AgNPs2, CHI‒AgNPs3, CHI‒AgNPs4, and CHI‒AgNPs5, is illustrated in Figure 3. The obtained results showed maximal LE of 92% in the conjugate (CHI‒AgNPs3) prepared with 3% AgNPs, followed by CHI‒AgNPs4 and CHI‒AgNPs2, i.e., 79% and 65%, respectively. Other tested concentrations showed the lowest LE, which could be due either to the lower concentration (1%) of AgNPs in the sample CHI‒AgNPs1 or the higher concentration (5%) of AgNPs in the sample CHI‒AgNPs5. At a lower concentration, there could be not enough particles available to develop an optimal conjugate, whereas a higher concentration may have caused unreacted particles to be washed away. The CHI‒AgNPs conjugates were further tested for their antibacterial, antibiofilm, and anticancer potential against *S. aureus*, *E. coli*, and *P. aeruginosa* and a breast cancer cell line, i.e., MCF-7.

### 3.4. Evaluation of Antibacterial Activity of CHI‒AgNPs Conjugates

**Figure 4:**
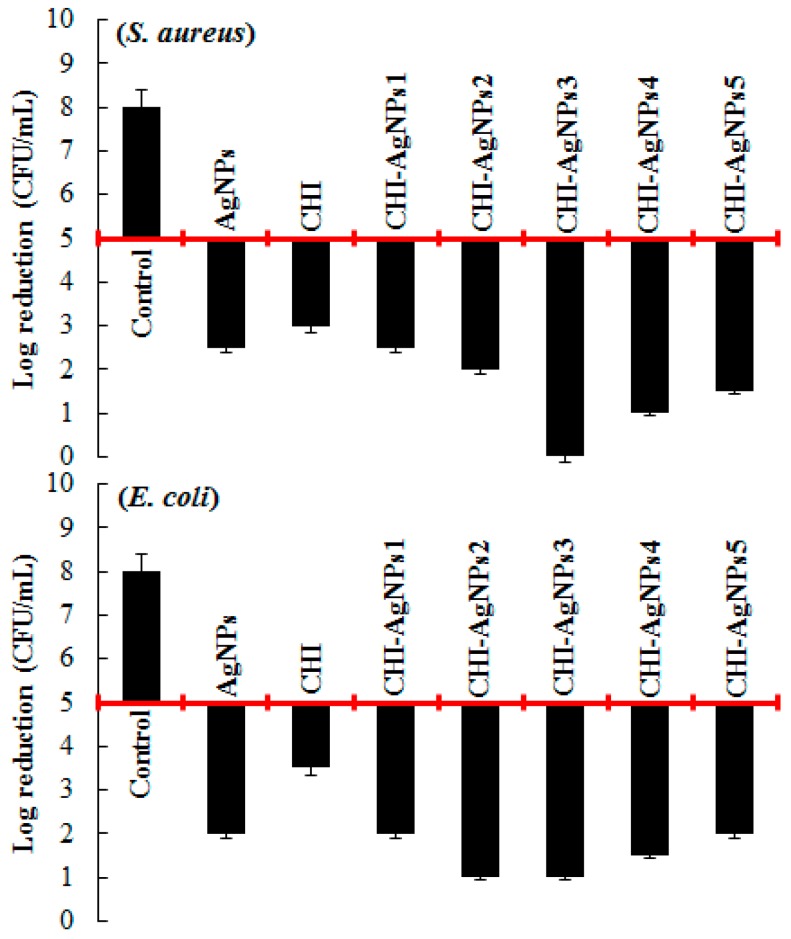
The antibacterial potentialities of pristine *Convolvulus arvensis* AgNPs and GA-assisted AgNPs loaded CHI‒AgNPs conjugates against the Gram-positive bacterial strain, i.e., *S. aureus* and Gram-negative bacterial strain, i.e., *E. coli*. Due to the intrinsic variability of the antibacterial test results, at least a 2-log reduction was considered necessary to claim antibacterial activity.

The antibacterial potentialities of pristine *C. arvensis* AgNPs and GA-assisted AgNPs-loaded CHI‒AgNPs conjugates were evaluated against the bacterial strains of *S. aureus* and *E. coli*. The results obtained are shown in Figure 4. *C. arvensis* AgNPs (alone) were found to be bactericidal up to a certain extent against all the tested strains. The optimally yielded CHI‒AgNPs3 conjugate was found to be highly bactericidal against both test strains. As shown in Figure 4, a maximal log value reduction from 5 to 0 against *S. aureus* and 5 to 1 against *E. coli* was recorded. CHI‒AgNPs2 and CHI‒AgNPs4 showed log reduction from 5 to 1 against *E. coli* and *S. aureus*, respectively. Based on the literature data, various mechanisms, i.e., DNA damage, cell membrane disruption, ROS, are used to explain the antibacterial potential of AgNPs [62,63]. However, the antibacterial activity depends on the size and dose of AgNPs. Ag^+^ ions interact with sulfur- or phosphorus-containing groups of proteins available in the bacterial cell wall or plasma membrane cause cytoplasmic fluids to leak out of the cell, which ultimately leads to bacterial cell death [50,64].

### 3.5. Antibiofilm Activity of CHI‒AgNPs Conjugates

**Figure 5:**
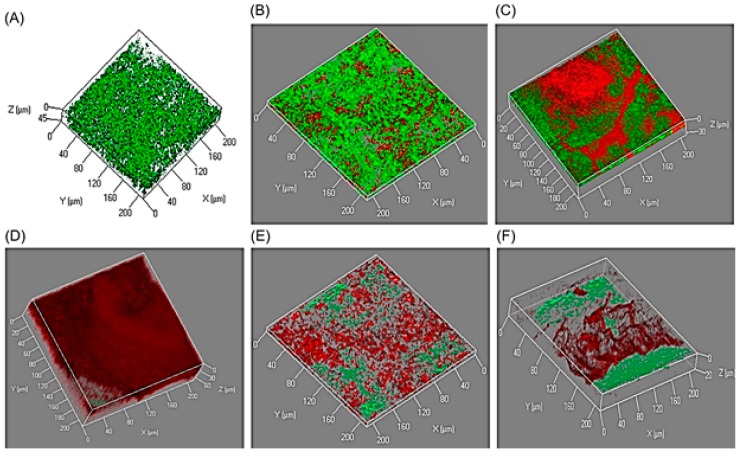
Antibiofilm activity of CHI‒AgNPs conjugates against *Pseudomonas aeruginosa*: (**A**) control, (**B**) CHI‒AgNPs1, (**C**) CHI‒AgNPs2, (**D**) CHI‒AgNPs3, (**E**) CHI‒AgNPs4, and (**F**) CHI‒AgNPs5.

AgNPs and/or AgNPs-based novel materials are considered multifaceted potential candidates for biomedical applications at large. Owing to their high surface/mass ratio along with significant antibacterial activity, AgNPs or AgNPs-based novel materials can preferably be applied as layers at the surface to avoid bacterial biofilm formation, which is a crucial pathogenic mechanism of several bacterially associated infections. In this context, the antibiofilm potentialities of newly developed CHI‒AgNPs conjugates were tested against *P. aeruginosa*. As shown in Figure 5, CHI‒AgNPs3 was the most active conjugate, in which significantly minimal survivability of *P. aeruginosa* was recorded as compared to the control sample value. As expected, this finding also corresponds to and confirms the antibacterial activity. This could be due to Ag^+^ interactions from the AgNPs and CHI‒AgNPs conjugates with DNA or available proteins in the bacterial cell wall, which ultimately lead to cell death [54,65,66]. To further confirm the influence of AgNPs-loaded CHI‒AgNPs conjugates on the tested strain viability, all test conjugates were studied by CLSM. The images recorded from the CLSM are shown in Figure 5. A high intensity of red fluorescent cells (dead cells) was observed when they were incubated with the CHI‒AgNPs3 conjugate. In samples from other conjugates, i.e., CHI‒AgNPs2 and CHI‒AgNPs4, the bacterial cells in closer proximity to the conjugate surfaces were found dead (red), while those away from the conjugate surfaces were able to survive, as shown by green fluorescent cells (live cells). Thus, the data obtained herein showed that AgNPs and CHI‒AgNPs conjugates impede the biofilm formation of *P. aeruginosa*. Earlier studies have shown that different concentrations of AgNPs for 48 h in a 96-well plate impair the biofilm formation of *P. aeruginosa* [67]. Likewise, in another study, Chaudhari et al. [68] reported the effect of biosynthesized AgNPs on *Staphylococcus aureus* biofilm quenching and prevention of biofilm formation.

### 3.6. Cytotoxicity Analysis of CHI‒AgNPs Conjugates

**Figure 6:**
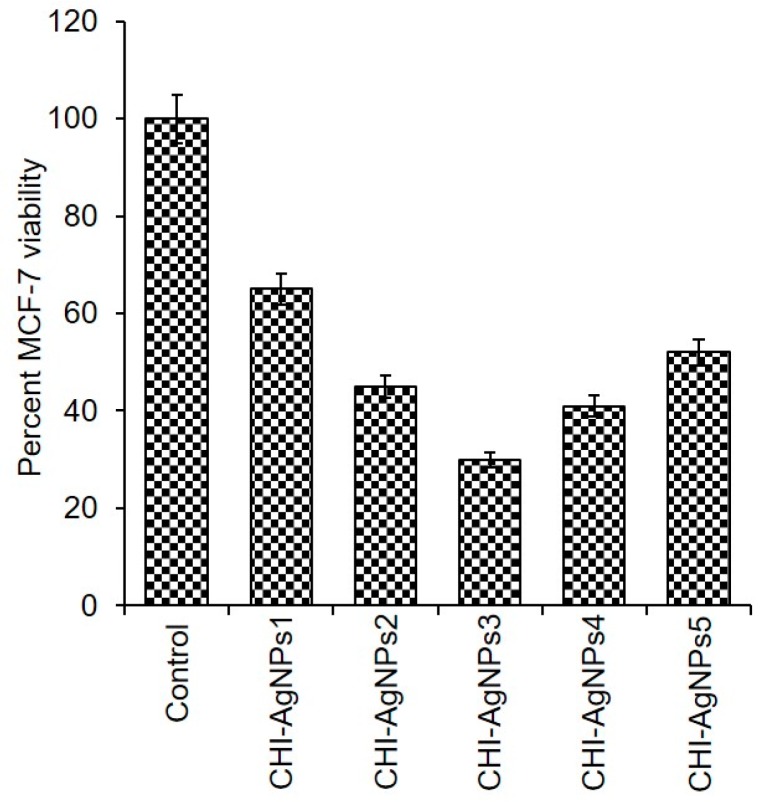
Percent viability of newly developed CHI‒AgNPs conjugates against breast cancer cell line, i.e., MCF-7.

The cytotoxicity of pristine AgNPs from different sources is well reported. However, there is not much data available on the cytotoxicity of AgNPs-loaded conjugates against the MCF-7 breast cancer cell line, which hold notable potentialities to be exploited for different biomedical applications such as against human carcinoma, e.g., breast and skin cancer, etc. To further strengthen this research, we studied the anticancer potentialities of newly developed CHI‒AgNPs conjugates against MCF-7 cells. In the presence of CHI‒AgNPs conjugates, the MCF-7 cells were grown in 96-well microtiter plates at 37 °C. The cytotoxic profile of CHI‒AgNPs conjugates in terms of percent cellular viability is shown in Figure 6. Interestingly, the pristine AgNPs were less effective than their CHI-loaded conjugates. Compared to the control sample, the optimally yielded CHI‒AgNPs conjugates result in lower cell viability for MCF-7 cancerous cells. Further to this, the 50% inhibitory concentration value of tested CHI‒AgNPs conjugates against MCF-7 cells was achieved within 48 h of incubation. The recorded inhibitory concentration and cell death/viability rate indicate the anticancer potential of CHI‒AgNPs conjugates. In an earlier study, Qi and Xu [69] synthesized chitosan-based NPs as potential anticancer agents and evaluated them in vitro against the Sarcoma-180 and mouse hepatoma H22 cancer cell lines. Multiple anticancer mechanisms responsible for chitosan-based NPs have been reported that effectively inhibit the proliferation of the human carcinoma cell line in vitro [70,71,72] and may be a beneficial agent against human carcinoma.

## 4. Conclusions

In conclusion, we report a biogenic synthesis of AgNPs using a freshly prepared extract of *C. arvensis* leaves. The optimally yielded reaction conditions were used to extract/purify *C. arvensis* AgNPs and used to develop GA-assisted CHI‒AgNPs conjugates. The newly engineered GA-assisted CHI‒AgNPs conjugates with bioactive potentialities were found to be promising for biomedical applications. The optimally yielded conjugate, i.e., CHI‒AgNPs3 with 92% LE, showed the highest level of antibacterial, antibiofilm and anticancer activity in the *S. aureus*, *E. coli*, and *P. aeruginosa* and MCF-7breast cancer cell line. In summary, based on the results obtained, we conclude that the newly engineered AgNPs and CHI‒AgNPs conjugates could be useful candidates for biomedical applications and are not harmful towards the environment.
